# Exploring the impact of psychological empowerment on customer orientation through psychological safety: the role of professional commitment as a moderator

**DOI:** 10.1186/s40359-025-03345-0

**Published:** 2025-09-26

**Authors:** Wan-Hsien Hu

**Affiliations:** School of Economics and Management, Sanming University, No. 25, Jingdong Rd., Sanyuan District, Sanming, Fujian 365004 China

**Keywords:** Customer orientation, Psychological empowerment, Psychological safety, Professional commitment

## Abstract

**Background:**

This study, grounded in the Conservation of Resources (COR) theory, presents a framework designed to explore the direct impact of psychological empowerment (PE) on psychological safety (PS) and its indirect influence on customer orientation (CO), all while considering the moderating role of professional commitment (PC).

**Methods:**

Using a convenience sampling method, this study targeted frontline employees working in star-rated hotels in China. Data were collected in two waves, resulting in a total of 189 valid responses. The collected data were then analyzed using Partial Least Squares Structural Equation Modeling (PLS-SEM) to test the hypothesized relationships among PE, PS, CO, and PC.

**Results:**

The analysis revealed a positive relationship between PE and CO, which is mediated by PS. Furthermore, the moderating effect of PC was supported: the indirect effect of PE on CO via PS is stronger when PC is high, and significantly weaker when PC is low. This finding highlights the importance of PC in enhancing the influence of PE on CO.

**Conclusion:**

This study not only proposes both theoretical and practical implications based on these findings but also discusses the limitations of the research and suggests potential directions for future studies.

## Introduction

Organizations are increasingly transitioning away from traditional hierarchical control structures in favor of a more empowered approach [1], which places particular emphasis on the importance of empowerment within service industries [2]. For example, Memon et al. [3] suggested that empowerment in the hospitality industry is a key predictor of front-line employees’ customer-oriented voice behavior. On the one hand, this shift toward empowerment enables managers to allocate more time to address urgent issues and strategic initiatives. On the other hand, employees who perceive a sense of empowerment in their roles experience greater meaning and purpose in their work, enhance self-efficacy, and increase competence. These positive experiences foster intrinsic motivation, leading to improved performance outcomes [4].

Regardless of whether the focus is at the individual or team level, scholars have conducted extensive research exploring the connections between PE and employees’ various attitudes and behaviors. The effects of PE extend to include both in-role and extrarole performance [1], as well as organizational commitment [1, 5]. Additionally, PE has been linked to intrinsic motivation, employee creativity [6], and overall job satisfaction [7]. In their comprehensive meta-analysis, Seibert et al. [7] provided an in-depth exploration of PE and its nomological network, further illustrating the multifaceted implications of empowerment in organizational contexts.

Researchers have called for a deeper examination of how empowerment affects PS (e.g., [8]), particularly in the hospitality industry, where intensive employee–customer interactions demand resilience. PS is crucial for employees managing both internal and external uncertainties within organizations [9]. Despite extensive studies, the causal relationship between PE and PS remains unclear. For example, Dutch health organizations’ qualitative evidence has shown that structural empowerment increases organizational resilience, but only if it is supported by PS and consistent top management commitment [10]. While some studies propose that PS enhances PE by encouraging individuals to contribute [11, 12], others argue that PE fosters PS, as empowered employees perceive their environments as safer [13]. Additionally, research has explored PE and PS as parallel mediators, examining their roles in influencing outcomes (e.g. [14–16],). These varied findings underscore the complexity of the PE-PS relationship and the need for greater clarity.

Frazier et al. [17] and Simonet et al. [12] emphasized that both PE and PS represent states of freedom within the workplace, motivating individuals to invest their ideas, initiatives, and efforts. The key distinction is that PE reflects an individual’s perception of influence and agency in their role [4, 12], whereas PS pertains to interpersonal dynamics in expressing ideas and taking action within a collaborative environment and how individuals perceive others’ responses to risk taking [12, 17, 18]. Thus, PE fosters self-initiative and accountability, whereas PS focuses on behaviors in the presence of others, creating a sense of safety and support for open communication [12]. Building on social cognitive theory, Simonet et al. [12] proposed that PS positively influences PE by creating a safe psychological environment that can empower individuals to engage in free expression; however, they noted a limitation in their cross-sectional data, which prevented causal claims and raised the possibility of reverse causality, where PE might also influence PS.

Drawing upon the COR theory [19], this study posits that PE positively influences PS. Ben-Zur and Yagil [20] suggested that empowerment serves as a crucial organizational resource that can effectively mitigate the emotional exhaustion experienced by employees. Although their research did not specifically differentiate among various types of empowerment—such as psychological versus structural empowerment—they utilized Spreitzer’s [21] definitions and measurement tools for PE. This choice implies that Ben-Zur and Yagil [20] regarded PE as a significant organizational resource. Sahadev et al. [22] explicitly suggested that PE is a personal resource that could enhance salespeople’s creative performance. When employees perceive their work as meaningful and autonomous, they are more likely to develop novel ideas and explore new approaches to performing existing tasks. Moreover, employees who feel PS are more likely to share innovative ideas and suggest improvements that benefit the organization and customers. This proactive attitude is especially important in the dynamic and high-pressure hospitality industry [23]. Therefore, frontline employees require these resources to effectively meet the needs of their customers.

However, despite the growing body of research on PE and PS, a significant gap remains in the literature regarding a comprehensive examination of the outcomes and contextual moderators of PE, particularly within the hospitality industry. For example, a review by Memon et al. [3] suggested examining the relationship between PS and the CO of frontline employees in the hospitality industry in high power-distance countries. Studies have focused on the relationships between PE and PS (e.g., [13]) or between PE and CO (e.g., [24, 25]) but have overlooked how individual moderators, such as PC, influence these relationships. In the hospitality industry, CO represents a critical aspect of service effectiveness and overall performance, whereas PC reflects an individual’s attachment to and identification with their profession [26, 27]. High turnover rates and labor shortages have long been persistent challenges for hospitality operators. As a result, it is crucial for both researchers and practitioners to focus on enhancing hospitality employees’ commitment to their profession [28]. PC serves as a significant determinant of employee work behavior, functioning as an essential individual difference that goes beyond mere personality traits and acts as a valuable personal resource [29, 30]. Drawing on COR gain spirals, this study introduces a framework for investigating the direct impact of PE on PS as well as exploring its indirect influence on CO through PS, with a focus on the moderating role of PC among frontline hotel employees.

Taken together, owing to the characteristics of face-to-face service immediacy and pressure in the hospitality industry, the direct path from PE to CO has gradually garnered attention. However, the underlying mechanisms of this relationship still require further detailed investigation. This study provides two significant contributions to the literature. First, building on COR gain spirals, this study clarifies the intricate relationship between PE and PS while also examining how these factors collectively influence CO. This clarification is essential for understanding the underlying mechanisms driving employee performance in the hospitality industry [3]. Second, this work posits that individuals who possess greater resources are better equipped to leverage these assets to achieve their work-related goals. In this context, PC serves not only as an individual trait resource but also as a source of variation among individuals, highlighting its role in influencing employee behavior and outcomes [31]. Consequently, this study explores and empirically tests the hypothesis that PC may serve as a moderating variable in the relationships among PE, PS, and CO.

**Fig. 1:**
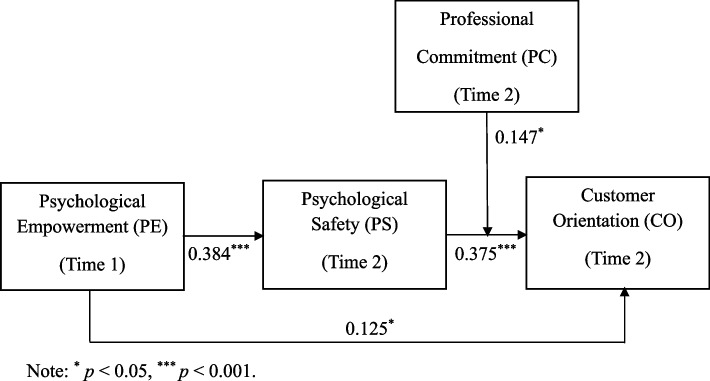
Research framework

The research framework is illustrated in Fig. 1.

## Literature review and hypothesis

### Conservation of Resources (COR) Theory 

The COR is one of the theoretical frameworks commonly used to explore the antecedents of CO [3]. Resources are defined as “those entities that either are centrally valued in their own right or act as a means to obtain centrally valued ends” [32] (p. 307). COR theory asserts that when these valuable resources are lost, a net loss occurs—this is characterized by a situation where the number of lost resources exceeds any resources that may have been gained. Furthermore, when prior investments in resources fail to generate new resources, stress levels increase among individuals [19, 33]. Individuals must allocate resources to prevent resource loss, recover from losses, and acquire new resources [33].

COR theory emphasizes that, although resources can be depleted over time, they can also be supplemented and replaced, thus allowing for recovery and growth [19, 33]. According to Hobfoll and Shirom [34], individuals who have access to a greater quantity of resources or superior resource pools are generally less susceptible to the adverse effects of resource loss. Moreover, these individuals may effectively utilize their existing resources to acquire additional resources, creating a beneficial gain spiral. Conversely, the loss of resources can lead to a detrimental loss spiral, where the absence of resources perpetuates further deprivation [33]. In other words, when employees have resources at their disposal, they can leverage these assets to generate or acquire new resources, thereby enhancing positive outcomes [35]. Edmondson [36] highlighted that, in addition to structural features within an organization, various resources can also promote PS; PE is one such essential resource [22]. Drawing on the concept of resource gain spirals [34], this resource can facilitate the generation of another resource, namely, the PS.

### Relationship between PE and CO

Empowerment is a process that identifies and addresses the factors contributing to employee powerlessness in the context of task completion. By systematically eliminating these barriers, organizations can significantly increase employee self-efficacy [37]. Building upon the foundational work of Conger and Kanungo [37], Thomas and Velthouse [38] defined PE as a form of intrinsic motivation that is articulated through four distinct cognitive dimensions. These dimensions collectively reflect individuals’ orientations and attitudes toward their work roles. These dimensions are competence, self-determination, meaning, and impact [21]. Competence refers to self-efficacy at work, i.e., the belief in one’s ability to utilize one’s skills to accomplish job tasks; self-determination pertains to one’s autonomy to decide what and how to perform one’s work role, including the ability to make decisions regarding methods, pace, and effort in the context of work tasks; meaning refers to individuals’ subjective judgments regarding the value of their job; and finally, impact refers to the extent of an individual’s effort at work, which can influence strategic, administrative, or operational outcomes [7, 21].

Employees’ perceptions of empowerment are often divided into psychological and structural empowerment (e.g., [7, 39]). The difference lies in the fact that structural empowerment focuses on workplace conditions [40], whereas PE concerns employees’ perceptions of those conditions. PE refers to an individual’s internal psychological experience of intrinsic motivation at work and, thus, reflects positive attitudes and feelings toward one’s work role, including the desire to positively impact the work environment through self-adjustment [4]. However, structural empowerment involves access to information, support, power (both formal and informal), and sufficient resources and opportunities to promote learning and growth in the job [41, 42]. In short, PE emphasizes individuals’ cognitive and motivational aspects related to power within the organization [4, 37, 38]; it represents the positive experiences individuals may perceive when they are fulfilled across four dimensions—competence, self-determination, meaning, and impact—in their work [4, 38].

CO pertains to the variations in employees’ attitudes toward service delivery, allowing them to address customer needs in a way that aligns with the specific situations of those customers [43]. CO also refers to a behavioral phenomenon that is embedded within the concept of marketing, which aims to incentivize employees in service organizations to provide better service to customers [44]. In other words, CO is both an attitude-related tendency and a behavioral manifestation. Specific examples include attempting to help customers achieve their goals, discussing customer needs, or influencing customers through information rather than pressure [45]. Joshi and Randall [46] suggested that, compared with service performance, CO is indicative of the long-term performance of service industries. Thus, CO serves as a performance indicator reflecting the extent to which employees internalize serving customers as a core value.

According to COR theory, when employees perceive a high level of PE—characterized by a sense of competence, self-determination, meaning, and impact in their work—they are more likely to view assisting customers as meaningful and feel empowered to serve them autonomously. This sense of empowerment not only enhances employees’ confidence in voicing their opinions to improve organizational outcomes [3] but also fosters the belief that they can influence key organizational policies, particularly those related to customer service. As a result, psychologically empowered employees are more likely to engage in extrarole behaviors, such as organizational citizenship behaviors (OCBs), which include helping behaviors (e.g., [47]) and voice behaviors (e.g., [48]). Customer-oriented behavior, a specific form of helping and voicing behavior, focuses on assisting customers and can be considered a type of customer-oriented OCB. For example, Auh et al. [49] argued that psychologically empowered frontline employees are more likely to engage in service-oriented citizenship behaviors because they perceive that going beyond their routine responsibilities significantly benefits customers. Previous studies have empirically supported the influence of PE on CO (e.g., [24, 25]). Taken together, PE enables employees to believe their job is meaningful and that they can make a difference while also enabling them to leverage their mastery and skills to help or speak up for customers. Accordingly, the following hypothesis is proposed:

Hypothesis 1: PE is positively correlated with CO.

### The mediating effect of PS

Kahn [15] described individual-level PS as the ability of employees to express their thoughts and feelings openly without worrying about negative impacts on their self-image, status, or career. Edmondson [36] highlighted the role of PS in team-level learning behavior. Early research on team learning focused on equipment, the physical environment, and pay systems rather than interpersonal factors. Edmondson [36] suggested that proactive learning behavior may put individuals at risk, as admitting that one needs assistance due to a lack of ability could damage one’s personal image, likelihood of promotion, and task assignments, and may even threaten one’s personal pride (i.e., face), thus hindering organizational learning and affecting employee engagement [50]. Therefore, Edmondson [36] argued for the establishment of a PS workplace in which individuals can experience interpersonal trust and mutual respect. More recently, Edmondson and Bransby [51] asserted that PS describes a work environment in which individuals believe that candor is both expected and possible. Additionally, Edmondson [36] examined PS in the workplace from the perspectives of image cost and saving face, emphasizing that these concepts are relevant not only in Western contexts but also in Chinese workplaces, highlighting the importance of PS across diverse cultural settings.

PE and PS both refer to positive motivational states toward work, but scholars have drawn clear distinctions between them. PE refers to individuals’ cognitive perceptions of specific tasks or responsibilities [17], whereas PS focuses on broader perceptions of the social and work environment, including how individuals perceive others’ reactions to taking risks in the workplace. Therefore, PS pertains to perceptions of the work environment rather than to specific tasks or responsibilities. PE can increase individuals’ enthusiasm and proactivity at work, thereby increasing job satisfaction, performance, and organizational competitiveness. When employees perceive that they are highly empowered, they are more motivated to explore effective work methods [4], engage in discussions with their supervisors and colleagues regarding work-related issues [38], and fulfill their job responsibilities more actively [4]. Additionally, empowered employees experience reduced fear of workplace changes and fluctuations and exhibit greater self-efficacy [37]. In rapidly changing environments, employees can grow, learn, contribute, and perform positively without worrying about damaging their personal image or status. Thus, when PE is high, employees feel safer and less concerned about potential consequences when expressing their opinions and ideas.

Research on organizational team behavior has shown that PS precedes variables such as speaking up as well as both team and organizational learning [52]. Furthermore, PS is a crucial antecedent of individual behaviors, including OCB and information sharing. The results of the meta-analysis conducted by Frazier et al. [17] supported the relationship between PS and OCB. CO can be defined in terms of employees’ helping behavior toward customers or identified as a form of extrarole behavior that is oriented toward customers. Previous research has indicated that both CO and OCB are forms of extrarole behavior [53]. According to the review article by Edmondson and Bransby [51], the service sector (e.g., health services and hospitality) frequently examines PS, likely because these industries must meet the diverse needs of customers. Sufficient PS is essential for making customer-oriented decisions. Edmondson and Bransby [51] also suggested that PS contributes to employees’ effectiveness in terms of “getting things done” (p. 61) because employees can offer upward feedback, share ideas, ask questions, and facilitate communication and coordination. For example, Wang et al. [54] suggested that when PS is present in the workplace, employees are more likely to feel capable and willing to engage in internal influencing behaviors, such as offering new perspectives on customer needs and contributing to potential improvements within tourism organizations. Gazzoli et al. [55] suggested that resources could be essential for encouraging certain employee citizenship behaviors. Therefore, it can be inferred that PS contributes to employees’ effectiveness in the context of serving customers.

According to COR theory, when employees perceive empowerment as a resource, their sense of PS as another resource is enhanced. Specifically, employees who exhibit strong perceptions of self-determination, competence, meaning, and impact in their work tasks are less concerned about seeking assistance from colleagues or supervisors to help customers, as they do not fear damaging their image or interpersonal relationships. Cultural factors such as high power distance and collectivism influence Chinese individuals to avoid conflict, preserve face, and prioritize relationship maintenance [56]. PS is essential for them to take initiative or engage in risky behaviors without fear of “losing face” [36, 56]. When they feel PS, they are more likely to direct their attention toward customers. In other words, PE enhances employees’ PS in the workplace [13]. When employees exhibit willingness, ability, and influence, they experience more PS and believe that additional resources can help them serve customers without fear of questioning, retaliation, or criticism from colleagues or leaders, thus leading them to engage in extrarole behavior. Therefore, this study hypothesizes that PE indirectly enhances CO through PS and proposes the following hypothesis:

Hypothesis 2: The relationship between PE and CO is mediated by PS.

### The moderating effect of PC

PC refers to the degree to which employees feel connected to and engaged with their profession [29]; more specifically, it reflects individuals’ attachment to and identification with their profession [26] and is a crucial factor in employees’ work behavior [29]. According to Blau’s [57] definition, career commitment is “one’s attitude toward one’s profession or vocation” (p. 278). Therefore, numerous terms, such as occupational commitment, career commitment, and career salience, are similar to PC. Common to all these terms is the critical notion of being committed to one’s career or occupation [58]. While the notion of PC originated in the field of industrial and organizational psychology, it was subsequently introduced into research on professionals and professional service firms, such as those in the financial accounting industry (e.g., [59]). Owing to the rise of the hotel service industry and the specialization of labor, PC has also been applied to employees working in hotel services. For example, an empirical study by Zhou et al. [60] demonstrated that the effect of organizational socialization on employee retention via person–environment fit was moderated by career commitment in the hotel industry.

When individuals demonstrate a high level of PC, they are willing to invest more effort into their work, accept professional goals, and engage in positive interactions with colleagues in the workplace. Even in negative environments (such as perceived organizational politics), such employees are willing and able to share knowledge actively with their colleagues [61]. Previous studies on PC have focused mainly on contexts such as health care (e.g., [62]), social work (e.g., [63]), and education (e.g., [64]), some of which are regarded as service industries that involve emotional labor and are often associated with low pay and long working hours (e.g., [65]). Therefore, investigating PC among employees in the hospitality industry is highly relevant.

When employees exhibit a higher level of PC, this indicator signifies that they are more willing to be deeply integrated into their profession and identify with their work more strongly. Therefore, under conditions featuring high levels of PC, the perceived indirect effect of PE on CO through PS is stronger. Specifically, in the hospitality industry, PC is strongly correlated with providing high-quality service to customers. Moreover, when employees feel psychologically safe and highly committed to their profession, they are more willing to meet customers’ needs and more capable of doing so. Thus, this study presents the hypothesis that PC effectively moderates the second stage of this mediated relationship. Accordingly, the following hypothesis is proposed:

Hypothesis 3: The indirect effect of PE on CO via PS is moderated by PC.

## Method

### Sample and procedure

This study included the voluntary assistance of three hotel interns in collecting data via convenience sampling and designing an online questionnaire with an informed consent form (via Wenjuanxing, www.wjx.cn). The specific approach involved contacting interns at star-rated hotels in Jiangsu Province to help identify frontline employees (colleagues) at these hotels, who voluntarily completed the questionnaire as respondents. To mitigate the potential impact of common method variance on the research results, the questionnaire distribution was divided into two waves. The first wave measured PE, whereas the second wave measured PS, PC, and CO. The two waves were separated by an interval of approximately two weeks.

To ensure the confidentiality of the participants’ personal information, all the responses to the online questionnaire were collected anonymously. However, participants were requested to provide the last four digits of their mobile phone numbers separately in each of the two waves of data collection. This information was necessary to facilitate the accurate pairing of data from the questionnaires while maintaining participant anonymity. Ultimately, a total of 200 valid questionnaires were collected across the two waves of research, and after the pairing process, 189 questionnaires remained valid for analysis. This careful approach to data collection and pairing underscores our commitment to protecting participant privacy while ensuring the integrity of the research findings.

The demographic data reveals a sample comprising 46 males (24.34%) and 143 females (75.66%). In terms of age, the majority fall within the 21–25 years category, accounting for 84 individuals (44.45%), followed by 45 participants (23.81%) aged 26–30 years. The age distribution includes 21 participants (11.11%) between 31 and 35 years, 23 individuals (12.17%) aged 41–45 years, 8 participants (4.23%) under 20 years, 7 individuals (3.70%) between 36 and 40 years, and a single participant (0.53%) aged 46–50 years. Regarding education levels, most respondents hold a university or college degree (114, or 60.32%), while 53 (28.04%) completed senior high school, 19 (10.05%) attended junior high school, and 3 (1.59%) have graduate school education.

### Measurement

Except for CO, which was measured using a Likert-5 scale (1 = strongly disagree, 5 = strongly agree), all other variables were measured using a Likert-7 scale (1 = strongly disagree, 7 = strongly agree). As shown in Table 1, all Cronbach’s alphas exceed 0.700.

**Table 1:** Means, standard deviations, correlations and HTMT results

Variables	M	SD	PE	PS	PC	CO
1. Psychological Empowerment (PE)	4.64	0.91	—	0.512	0.236	0.443
2. Psychological Safety (PS)	4.92	1.22	0.384^**^	—	0.625	0.833
3. Professional Commitment (PC)	4.68	1.17	0.014	0.513^**^	—	0.762
4. Customer Orientation (CO)	3.83	0.69	0.258^**^	0.662^**^	0.630^**^	—

*n* = 189, ^**^ p < 0.01. Below the diagonal elements are the correlations values. Above the diagonal elements are the HTMT values

#### PE

Employing a Chinese scale modified by Li et al. [66] and originally developed in English by Spreitzer [4], this scale comprises a total of 12 items organized across four dimensions. The dimensions consist of meaning (3 items), such as: “The work I do is very important to me”; self-determination (3 items), illustrated by: “I have significant autonomy in determining how I do my job”; self-efficacy (3 items), exemplified by: “I am confident about my ability to do my job”; and impact (3 items), with an example being: “My impact on what happens in my department is large.”

#### PS

Utilizing a Chinese scale developed by Li and Yen [67], which was adapted from the original scale developed by May et al. [50], comprising a total of five items. Among these, four items are reverse scored, with example items such as: “I’m not afraid to be myself at work” and “There is a threatening environment at work” (reverse-scored item).

#### PC

Using the measurement developed by Suddaby et al. [59], comprising a total of seven items. An example item as: “This job is a significant part of my work life”.

#### CO

Using the measurement developed by Homburg and Stock [45], comprising a total of five items. An example item as: “I try to help the customers to achieve their goal”.

### Analysis strategy

This study examined the conditional indirect effect of PE on CO through PS at different levels of PC. Given the small sample size (less than 200), partial least squares regression (PLS) is more appropriate for this study. Therefore, SmartPLS 4 [68] and SPSS were used to analyze the data.

## Result

### Measurement model evaluation

This study considers SmartPLS to test hypotheses [68]. The effect sizes for all hypotheses were assessed using the bootstrapping technique with 5,000 resamples. Table 1 presents descriptive statistics, correlation coefficients, and heterotrait-monotrait (HTMT) ratio of correlations. For discriminant validity criteria, all HTMT ratio values are less than 0.850 [69]. Table 2 presents the construct validity and reliability. Although the AVE value of PE was marginal (AVE = 0.485), composite reliability value was above the acceptable threshold level.

**Table 2:** Construct validity and reliability

	Cronbach’s alpha	Composite reliability (rho_a)	Composite reliability (rho_c)	Average variance extracted (AVE)
PE	0.906	0.917	0.918	0.485
PS	0.799	0.846	0.859	0.555
PC	0.839	0.847	0.878	0.509
CO	0.803	0.825	0.860	0.513

### Common method variance test

Although data were collected using the time-lag method, all measurements were self-reported by employees. This could result in common method variance (CMV) impacting the results [70]. Therefore, Harmon’s one-factor test was used to address this issue. The unrotated factor analysis showed that the cumulative explained variance of all extracted factors was 62.297%, with the variance of the first factor being 28.412%, which did not account for more than 50% of the cumulative explained variance [71]. This indicates that the effect of common method variance is limited.

### Hypotheses testing

The results of path coefficients were shown in Fig. 1. The R^2^ for the endogenous variable (i.e., CO) is 0.574 (t = 11.434, *p* < 0.001, 95% CI = [0.478, 0.672]) that represents a moderate explanatory power [72].

According to Hypothesis 1 (H1), there is an expected positive correlation between PE and CO. As shown in Table 3, PE exerts a significant positive effect on CO (*β* = 0.125, *p* < 0.05, 95% CI = [0.008, 0.243]), thus providing support for Hypothesis 1.

**Table 3:** Path analysis

Hypothesis	Path coefficients	Standard deviation	T-statistics	*P*-values	95% CI
H1: PE ➜ CO	0.125	0.059	2.123	0.034	[0.008, 0.243]
H2: PE ➜ PS ➜ CO	0.144	0.035	4.138	0.000	[0.082, 0.217]
PS⨯ PC ➜ CO	0.147	0.060	2.459	0.014	[0.036, 0.266]

Hypothesis 2 (H2) posits that the relationship between PE and CO is mediated by PS. As shown in Table 3, the indirect effect of PE on CO via PS is 0.144 (*p* < 0.001, 95% CI = [0.082, 0.217]). Therefore, Hypothesis 2 is supported.

**Fig. 2:**
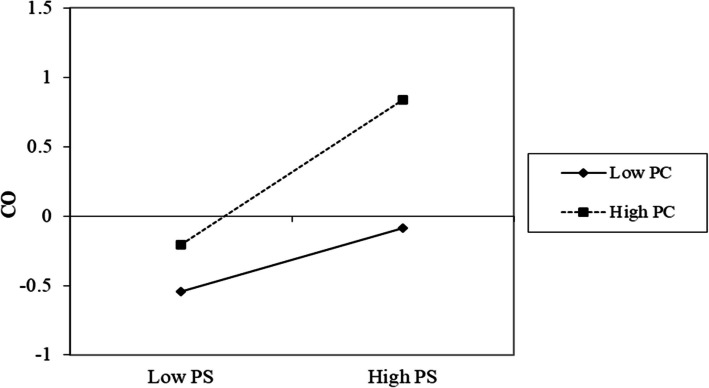
PC’s moderating effect on the relationship between PS and CO

Hypothesis 3 (H3) articulates the expectation that the indirect effect of PE on CO through PS will be moderated by PC. As shown in Table 3 reveals that the relationship between PS and CO is moderated by PC (*β* = 0.147, *p* < 0.05, 95% CI = [0.036, 0.266]). Figure 2 illustrates that PS had a stronger positive relationship with CO when perceived PC was strong. Next, the moderated mediation effect (5000 bootstrapping) reveals a significant moderating effect of PC. As shown in Table 4, at high levels of PC, PE exerts a positive indirect effect on CO via PS, with an effect size of 0.200 (SE = 0.044, *p* < 0.001, 95% CI = [0.120, 0.294]); whereas at low levels of PC, this indirect effect weakens but remains significant, with an effect size of 0.088 (SE = 0.040, *p* < 0.05, 95% CI = [0.013, 0.169]). Therefore, Hypothesis 3 is supported.

**Table 4:** The results of moderated mediation analysis (H3)

Mediator	Moderator: PC	*β*	SD	T-statistics	*P*-values	95% CI
PS	+ 1SD	0.200	0.044	4.526	0.000	[0.120, 0.294]
	-1SD	0.088	0.040	2.186	0.029	[0.013, 0.169]

## Discussion and conclusion

Using the COR theory, this study explores the indirect effect of PE on CO through PS. PC—a specific resource held by employees—moderates this mediated relationship. Data were collected over two waves, resulting in 189 valid responses that provide empirical support for the proposed hypothesis. Building on previous research on PE and CO (e.g., [24, 25]), this study examines the mediating effect of PS and the moderating effect of PC in the hospitality industry. PS helps employees manage internal and external uncertainties within organizations [9], and PE, as both an organizational and personal resource, can enhance PS. Employees with high PS are better equipped to meet customer needs and deliver high-quality service in low-uncertainty environments. Furthermore, when there is a commitment to hospitality service professionalism, PE indirectly enhances CO through PS, leading to stronger outcomes.

### Theoretical implications

This study offers multiple theoretical contributions to the PE and PS literature, as well as their effects on CO.

First, based on COR theory, this study responds to Memon et al. [3] by examining the relationships among PE, PS, and CO in the hospitality industry. It also addresses Simonet et al.’s [12] concerns about the causal direction between PE and PS. Previous research has shown inconsistent findings regarding the relationship between PE and PS or has treated them as parallel mediators within the motivation process (e.g., [16]). By collecting data across two waves, this study reveals that PE leads to PS. From the perspective of PE as an organizational resource [20], it mitigates emotional exhaustion, whereas from the perspective of PE as a personal resource [22], it enhances performance. Both perspectives align with the COR theory’s concept of resource gain spirals [73], where resources not only prevent depletion but also lead to or generate new resources, thus indirectly improving performance. This study hypothesizes and tests that PE, as both an organizational and personal resource, enhances PS and indirectly improves CO in the hospitality industry. The findings align with those of Zhou and Chen [13]. However, unlike their study, this research focused on CO among hospitality employees and examined the moderating effect of individual characteristics, specifically PC, within this pathway. Additionally, the findings are consistent with those of Gazzoli et al. [55] and Yoo [48], who provide direct or indirect evidence for the impact of PE and PS on service orientation or employee voice directed toward customers. Employees will not act on structural empowerment opportunities unless they perceive themselves as empowered (i.e., PE) [40].

Second, Zhou et al. [60] noted that few studies have explored the role of PC in the hospitality industry. This study proposes that PC, which is based on a sample of hotel employees, is not only an important individual difference beyond the level of personality traits but also a resource possessed by individuals [30]. PC thus contributes to the availability of resources within the relationships among PE, PS, and CO and moderates these mediated relationships. This finding supports the notion of a resource pool posited by COR theory, given that individuals who already possess resources can invest these resources more effectively; furthermore, Halbesleben et al. [74] suggested that individuals with available resources have more opportunities to invest in additional resources. Since CO involves providing services that can meet customers’ needs both cognitively and emotionally, it may lead to resource depletion. Therefore, while relying solely on organizational or personal resources (PE and PS) can enhance CO, a high sense of commitment to service work can replenish the resources of employees who provide services. Therefore, this study broadens the application scope of COR theory.

### Practical implications

The results of this study provide a basis for proposing several management practice recommendations. First, previous research has suggested that frontline employees’ CO contributes to organizational success (e.g., [75]). Building upon this foundation, numerous studies have investigated the drivers of CO, while many studies have emphasized the importance of empowering employees to respond effectively to and fulfill diverse customer needs (e.g., [49, 76]). Since empowerment is a crucial resource in the workplace, human resource management (HRM) departments and leaders should cultivate a culture of empowerment to enhance employees’ sense of PE, thereby indirectly enhancing their customer-oriented behavior (e.g., [77]). Managers could focus on work design, leadership practices, and organizational support to psychologically empower their service employees (e.g., [22]). By prioritizing empowerment, organizations can better equip their employees to meet and exceed customer expectations. For example, delegating authority to frontline employees to respond to customers’ needs or complaints can foster a more responsive and customer-focused environment [78].

Second, within an environment characterized by a lack of PS, employees may perceive their circumstances as uncertain, unpredictable, and threatening [50]. To conserve their resources and minimize potential losses, such employees may reduce their CO. Even when such employees encounter challenges in customer interactions, they may refrain from seeking assistance or attempting to resolve issues, ultimately choosing to abstain from providing services altogether. Previous research has emphasized the importance of having supportive and trustworthy supervisors and colleagues in fostering a sense of PS [50]. For example, Lintanga and Rathakrishnan [79] reported that PS communication and feedback involve a reciprocal process in which management and employees share information, and this interaction enhances the overall sense of safety of the workplace. Gazzoli et al. [55] also suggested that organizations should create an environment where frontline employees feel “safe enough” to express their opinions (p. 10). Edmondson and Bransby [51] further highlighted that “ensuring that people can speak up, ask for help, offer ideas, provide dissenting views, or collaborate effectively across boundaries” is important (p. 72). Therefore, organizational leaders and HRM departments should prioritize the establishment and enhancement of a PS workplace environment. Providing frontline employees with opportunities for effective perspective-taking experiences can help achieve this goal [55]. By encouraging employees to proactively address service-related concerns and provide timely feedback, leaders and HRM departments can foster an environment that is conducive to increased PS and improved CO.

Third, PC plays a moderating role in enhancing the impact of PE on OC via PS. In HR practices in the hospitality industry, it is imperative to evaluate and prioritize candidates and employees who exhibit high levels of PC during recruitment and training processes. Moreover, assessing the PC of existing employees and intervening appropriately may enhance the moderating effect of employee PC. For example, managers could inform early-career employees about potential shifts in career attitudes and offer career planning support and skill development programs to highlight advancement opportunities in the hospitality industry. Building mentorships between newcomers and experienced employees on an upward career trajectory can also encourage positive career growth [28].

### Limitations and suggestions

Although this research has made several meaningful contributions to the literature, certain limitations must be recognized.

First, this study employed two waves of data collection to reduce CMV effects and could not fully eliminate the bias linked to the use of common source data. Although previous scholars have indicated that CMV may weaken rather than enhance interaction or moderation effects, the statistical testing of moderation effects is considered quite conservative [80]. Moreover, Harmon’s one-factor test suggests that CMV is limited, and future research is recommended to employ multiple methods for testing CMV to increase the rigor of the study.

Second, while this study provides valuable insights, the small sample size may limit the statistical power of the analyses and reduce confidence in the reported effects. Consequently, the generalizability of the findings should be interpreted with caution. Additionally, the study focused exclusively on star-rated hotels in Jiangsu, which limits the diversity of hotel categories included in the sample. As a result, the findings may not fully represent the broader spectrum of the hotel industry, particularly nonstar-rated hotels or those from different regions. Future studies should collect data from a broader range of hotel categories and use larger, more diverse samples to increase the statistical power and improve the robustness of the findings. A larger sample size would not only increase the generalizability of the research framework but also provide a more comprehensive understanding of the phenomena under study.

Third, this study focused solely on the individual-level perceptions of employees, thereby neglecting the potential influence of group-level or environmental factors. For instance, a meta-analysis by Schermuly et al. [81] demonstrated that various leadership styles—such as benevolent leadership, empowering leadership, and transformational leadership—can enhance perceptions of empowerment. Moreover, contextual factors such as strategic HRM can have a significant influence in this context [82]. Given these insights, future research could integrate leadership styles and HRM practices to examine their moderating effects within the research framework at both the individual and group levels.

Finally, the current study focused exclusively on PC as a trait variable of individual resources, neglecting other potential trait variables that could also play significant roles. For example, traits such as proactivity and extraversion may also serve as important moderators of the mediated effects proposed in this study. Therefore, future research should consider exploring a wider array of trait resources and their potential moderating effects on the relationships examined.

## Data Availability

The data that support the findings of this study are available on request from the corresponding author upon reasonable request.

## Abbreviations

CO: Customer orientation
COR: Conservation of Resources theory
PC: Professional commitment
PE: Psychological empowerment
PS: Psychological safety
